# Relationship Between Neoadjuvant Chemotherapy and Log Odds of Positive Lymph Nodes and Their Prognostic Role in Advanced Ovarian Cancer Patients With Optimal Cytoreductive Surgery

**DOI:** 10.3389/fonc.2022.878275

**Published:** 2022-05-16

**Authors:** Yue-min Hou, Yan Xue, Jin-meng Yao, Fang Feng, Rui-fang An

**Affiliations:** Department of Gynecology and Obstetrics, The First Affiliated Hospital of Xi’an Jiaotong University, Xi’an, China

**Keywords:** advanced ovarian cancer, neoadjuvant chemotherapy, optimal cytoreductive surgery, LODDS, prognosis, SEER

## Abstract

**Purpose:**

To analyze the relationship between neoadjuvant chemotherapy (NACT) and log odds of positive lymph nodes (LODDS), as well as their prognostic role in advanced ovarian cancer (AOC) patients with optimal cytoreductive surgery.

**Patients and Methods:**

From the SEER database during 2010-2016, we identified 1,012 AOC patients with optimal cytoreductive surgery. Univariable and multivariable logistic regression was performed to identify the relationship between NACT and LODDS. Overall survival (OS) and cancer-specific survival (CSS) were assessed using the Kaplan-Meier method and log-rank test. Univariable and multivariable Cox regression was conducted to determine the independent prognostic factors for OS and CSS.

**Results:**

Factors associated with significantly higher NACT odds included older (≥60 years old), married, tumor size ≥ 15 cm, FIGO IV, and LODDS ≤ 0.1. Multivariable Cox regression model confirmed older (≥60 years old), unmarried, separated, divorced, widowed, mucinous histology type, tumor size ≥ 15 cm, and LODDS > 0.1 were correlated with increased risks of OS and CSS. NACT was not an independent prognostic factor for OS and CSS. In the subgroup analyses, LODDS was an independent prognostic factor for OS and CSS in patients with < 75 years old, married, unmarried, FIGO III, and tumor size < 15 cm.

**Conclusion:**

NACT did not show any survival benefit in AOC patients with optimal cytoreductive surgery, but it may be beneficial in reducing LODDS. Meanwhile, clinicians can use grade of LODDS to predict the prognosis of AOC patients with optimal cytoreductive surgery.

## Introduction

Ovarian cancer (OC) is one of the most common malignant tumors and ranks as the fifth cause of cancer death among women in the United States (1). According to the latest cancer statistics, 13,770 patients died of OC, and 21,410 patients were newly diagnosed in the United States in 2021 (2). Given the nonspecific signs and symptoms of OC, such as abdominal bloating, abdominal pain, and unintentional weight loss, most patients are diagnosed at an advanced stage (3, 4).

Standard of care for patients with advanced ovarian cancer (AOC) is primary cytoreductive surgery (PCS) followed by adjuvant chemotherapy (5, 6). The leading prognostic factor in determining survival of patients with AOC is achievement of complete cytoreductive surgery with the aim of macroscopically complete resection of all visible tumor, followed by chemotherapy that includes carboplatin and paclitaxel (7). However, complete cytoreduction surgery may not be achievable in those with heavy peritoneal disease burden. An alternative management option in patients with AOC is initial treatment with neoadjuvant chemotherapy (NACT) to reduce tumor burden followed by interval cytoreductive surgery (ICS) (8). NACT-ICS offers several advantages over PCS, including an increased rate of optimal cytoreduction (no visible residual disease (RD) or RD ≤ 1 cm), lower perioperative morbidity, and higher quality of life in patients with an AOC who would likely not achieve optimal cytoreductive surgery (9, 10). The use of NACT has increased annually by 10.3% between 2011 and 2016 compared with an annual increase of 7.9% between 2006 and 2011 (11). But the clinical use of NACT is also controversial. Compared with PCS, NACT did not show any survival benefit in reported randomized controlled trials (RCT) or Meta analysis (10, 12–15). These results have led to concerns about the adverse effects of NACT.

In clinical practice, lymph node metastasis is one of the major metastases of AOC and an indicator for evaluating recurrence and survival, and a key prognostic factor in determining the development of postoperative treatment plans and follow-up (16). Traditionally, lymph node status is based on positive lymph nodes (PLNs) regardless of the number of resected lymph nodes (RLNs) (17). At present, the lymph node ratio (LNR), defined as the ratio between the number of PLNs and RLNs, is increasingly recognized as a powerful prognostic tool for many cancers (18, 19). The log odds of positive lymph nodes (LODDS) is the logarithm of the ratio of metastatic lymph nodes to negative lymph nodes (log (PLNs+0.5)/(RLNs-PLNs+0.5)) and can identify patients with highly homologous prognoses. It also overcomes the shortcoming of the LNR regardless of the number of lymph nodes examined (20). Recently, LODDS staging was hypothesized to be a better predictor of survival in many cancers compared with LNR and PLN staging (18, 21). Previously, LODDS was used to analyze the prognostic role of mesenteric lymph nodes (MLNs) involvements in AOC (22). However, the prognostic value of LODDS in AOC remains unclear. Therefore, the aim of the current study was to determine the most appropriate nodal staging system for OC patients with optimal cytoreductive surgery. Besides, no retrospective or prospective studies have been conducted to evaluate the relationship between NACT and LODDS. The Surveillance, Epidemiology, and End Results (SEER) cancer database, in the United States, collects information on 34.6% of Americans in 18 registries. Using the SEER database, we extracted details on AOC patients with optimal cytoreductive surgery between 2010 and 2016 to determine the prognostic role of NACT, LODDS, and the relationship between them. These findings will help doctors make better clinical decisions with individualized application of NACT.

## Materials and Methods

### Data Source and Study Population

**Figure 1:**
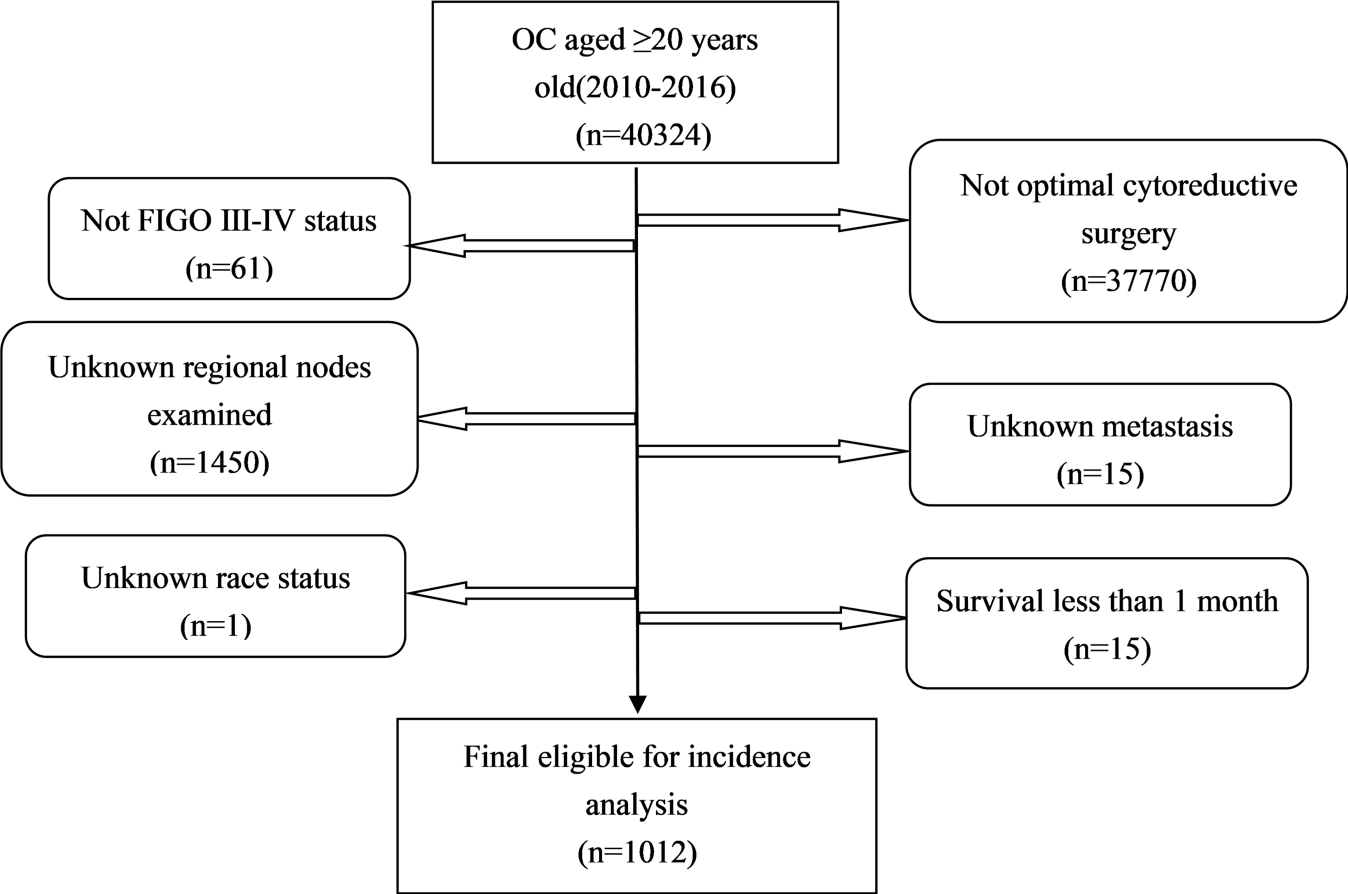
Flow chart of patient selection from the SEER database.

All the primary data were acquired from the SEER database. The SEER∗Stat version 8.3.9 (https://seer.cancer.gov/seerstat/) was used to screen eligible patients who were AOC (ICD-O-3, C56.9) with optimal cytoreductive surgery between 2010 and 2016. We gathered the following information: age at diagnosis, race, year of diagnosis, grade (G1 is equivalent to well differentiated; G2 is equivalent to moderately differentiated; G3 is equivalent to poorly differentiated; G4 is equivalent to undifferentiated), FIGO stage, marital status [other (separated, divorced, widowed)], laterality, Ca125, tumor size, metastasis site, RLNs, PLNs, LODDS, pathological subtype[Third Edition (ICD-O-3) morphology codes(8441/3, 8460/3, 8461/3 for serous; 8380/3 for endometrioid; 8310/3 for clear cell; 8480/3 for mucinous);], vital status, and survival time. The study calculated LODDS by log (PLNs+0.5)/(RLNs-PLNs+0.5). We added 0.05 to both the numerator and denominator to avoid an undefined number. LODDS was classified into LODDS1 (≤ 0.1) and LODDS2 (>0.1). Patients who were diagnosed by autopsy or death certificate with a follow-up shorter than 1 month were excluded. A total of 1,012 patients were eligible for incidence analysis. The primary endpoint was overall survival (OS) and secondary endpoint was cancer-specific survival (CSS). The OS was defined as the survival time in months regardless of the cause of death. The CSS was defined as the time interval from diagnosis of ovarian cancer to ovarian cancer -related death (**Figure 1**).

### Statistical Analysis

PCS and NACT-ICS were compared using the chi-square test (χ2). Univariable and multivariable logistic regression was performed to identify the relationship between NACT and LODDS. Odds ratios (OR) and 95% confidence intervals (CI) were reported from the logistic regression. Univariable and multivariable Cox regression analyses were employed to identify independent predictors associated with survival by reporting the hazard ratios (HR) and 95% CI. Survival comparisons were made using Kaplan–Meier analysis and log-rank tests.

Statistical analyses were all performed using SPSS (version 22.0, IBM Corporation, USA) and R software (version 3.6.3; www.r-project.org/). *P* < 0.05 (2-sided) was considered significant.

## Results

### Patient Characteristics

The demographic and clinical characteristics of the 1,012 patients are shown in **Table 1**. Most of them were white race (87.2%), married (55.0%), serous histologic type (80.8%), bilateral (66.9%), Ca125 positive (85.0%), grade3-4 (76.5%), FIGO III (70.8%), metastatic site of liver (6.1%), tumor size<15 cm (54.8%), LODDS ≤0.1 (67.0%). A total of 255 (25.2%) patients received NACT and 757 (74.8%) did not. There were an equal number of race, year of diagnosis, marital status, histologic type and bilateral in both PCS and NACT-ICS. Patients with older (≥60 years old), lower PLNs, lower LODDS, multiple sites of metastasis, tumor size ≥15 cm and FIGO IV were more likely to receive NACT.

**Table 1:** Baseline characteristics of AOC patients with optimal cytoreductive surgery.

	Total (N=1012)	PCS (N=757)	NACT-ICS (N=255)	*P*-value*
**Age**				**<0.001**
20-59	447 (44.2)	361 (47.7)	86 (33.7)	
60-74	443 (43.7)	313 (41.3)	130 (51.0)	
≥75	122 (12.1)	83 (11.0)	39 (15.3)	
**Race**				0.945
White	882 (87.2)	659 (87.0)	223 (87.5)	
Black	58 (5.7)	43 (5.7)	15 (5.9)	
Other	72 (7.1)	55 (7.3)	17 (6.6)	
**Year**				0.122
2010-2013	609 (60.2)	466 (61.5)	143 (56.1)	
2014-2016	403 (39.8)	291 (38.5)	112 (43.9)	
**Marital status**				0.212
Married	557 (55.0)	403 (53.3)	154 (60.4)	
Unmarried	212 (20.9)	165 (21.7)	47 (18.4)	
Other	208 (20.6)	160 (21.2)	48 (18.8)	
Unknown	35 (3.5)	29 (3.8)	6 (2.4)	
**Histology**				0.523
Serous	818 (80.8)	616 (81.4)	202 (79.2)	
Endometrioid	29 (2.9)	24 (3.2)	5 (2.0)	
Clear cell	25 (2.5)	18 (2.4)	7 (2.7)	
Mucinous	11 (1.1)	9 (1.2)	2 (0.8)	
Other	129 (12.7)	90 (11.8)	39 (15.3)	
**Laterality**				0.807
Unlateral	335 (33.1)	249 (32.9)	86 (33.7)	
Bilateral	677 (66.9)	508 (67.1)	169 (66.3)	
**Ca125**				**0.038**
Positive/elevated	860 (85.0)	632 (83.5)	228 (89.4)	
Negative/normal	23 (2.3)	21 (2.8)	2 (0.8)	
Unknown	129 (12.7)	104 (13.7)	25 (9.8)	
**Grade**				**<0.001**
1	16 (1.6)	12 (1.6)	4 (1.6)	
2	69 (6.8)	59 (7.9)	10 (3.9)	
3	389 (38.4)	303 (40)	86 (33.7)	
4	386 (38.1)	311 (41)	75 (29.4)	
Unknown	152 (15.1)	72 (9.5)	80 (31.4)	
**FIGO**				**<0.001**
III	717 (70.8)	577 (76.3)	140 (54.9)	
IV	295 (29.2)	180 (23.7)	115 (45.1)	
**Tumor size**				**0.040**
<15	555 (54.8)	426 (56.3)	129 (50.6)	
≥15	235 (23.3)	161 (21.3)	74 (29.1)	
Unknown	222 (21.9)	170 (22.4)	52 (20.3)	
**Metastasis**				**<0.001**
Bone	4 (0.4)	2 (0.3)	2 (0.8)	
Brain	1 (0.1)	1 (0.1)	0 (0.0)	
Liver	62 (6.1)	32 (4.2)	30 (11.8)	
Lung	51 (5.1)	30 (4.0)	21 (8.2)	
None	894 (88.3)	692 (91.4)	202 (79.2)	
**RLNS**	10.99 (1.00-85.00)	11.30 (1.00-85.00)	10.04 (1.00-73.00)	**<0.001**
**PLNs**	3.15 (0.00-49.00)	3.37 (0.00-49.00)	2.48 (0.00-43.00)	**<0.001**
**LODDS**				**0.029**
≤0.1	678 (67.0)	493 (65.1)	185 (72.5)	
>0.1	334 (33.0)	264 (34.9)	70 (27.5)	

*Bold P-value, P < 0.05.

### Determinants of NACT

**Table 2** shows distributions of patient characteristics according to NACT using univariable and multivariable logistic regression. On the multivariable logistic regression, compared to patients 20-59 years old, those 60-74 years old (OR:1.624, 95%CI: 1.173-2.257), ≥75 years old (OR:1.877, 95%CI: 1.192-3.020), and married patients (vs. unmarried patients, OR: 0.578, 95%CI: 0.381-0.861) had higher odds of NACT. Also, compared to patients with FIGOIII, those with FIGOIV (OR:2.854, 95%CI: 2.091-3.902) were associated with higher odds of NACT. Additionally, compared to tumor size <15 cm, tumor size≥15 cm (OR:1.450, 95%CI: 1.007-2.082) had higher odds of NACT. Further, patients with LODDS >0.1 (OR:0.589, 95%CI: 0.420-0.816) had lower odds of NACT compared to those with LODDS ≤0.1.

**Table 2:** Univariable and multivariable logistic regression for associations between patient characteristics and NACT.

Variable	Univariable		Multivariable	
	OR (95% CI)	*P*-value*	OR (95% CI)	*P*-value*
**Age**				
20-59	1 (Reference)		1 (Reference)	
60-74	1.743 (1.279-2.386)	**<0.001**	1.624 (1.173-2.257)	**0.004**
≥75	1.972 (1.254-3.073)	**0.003**	1.877 (1.159-3.014)	**0.010**
**Race**				
White	1 (Reference)			
Black	1.031 (0.545-1.851)	0.922		
Other	0.913 (0.505-1.574)	0.753		
**Year**				
2010-2013	1 (Reference)			
2014-2016	1.254 (0.940-1.671)	0.122		
**Marital status**				
Married	1 (Reference)		1 (Reference)	
Unmarried	0.666 (0.450-0.969)	**0.037**	0.578 (0.381-0.861)	**0.008**
Other	0.872 (0.602-1.250)	0.548	0.739 (0.500-1.080)	0.548
**Histology**				
Serous	1 (Reference)			
Endometrioid	0.635 (0.212-1.557)	0.363		
Clear cell	1.186 (0.455-2.765)	0.706		
Mucinous	0.678 (0.103-2.656)	0.621		
Other	1.321 (0.871-1.974)	0.181		
**Laterality**				
Unlateral	1 (Reference)			
Bilateral	0.963 (0.715-1.305)	0.807		
**Ca125**				
Negative/normal	1 (Reference)			
Positive/elevated	3.788 (1.100-23.802)	0.073		
**Grade**				
1	1 (Reference)			
2	0.508 (0.142-2.092)	0.314		
3	0.851 (0.288-3.106)	0.785		
4	0.723 (0.244-2.644)	0.584		
**FIGO**				
III	1 (Reference)		1 (Reference)	
IV	2.633 (1.954-3.549)	**<0.001**	2.854 (2.091-3.902)	**<0.001**
**Tumor size**				
<15	1 (Reference)		1 (Reference)	
≥15	1.518 (1.080-2.126)	**0.016**	1.450 (1.007-2.082)	**0.045**
**LODDS**				
≤0.1	1 (Reference)		1 (Reference)	
>0.1	0.707 (0.514-0.963)	**0.030**	0.589 (0.420-0.816)	**0.002**

*Bold P-value, P < 0.05.

### The Effects of LODDS and Different Treatment on OS and CSS

**Figure 2:**
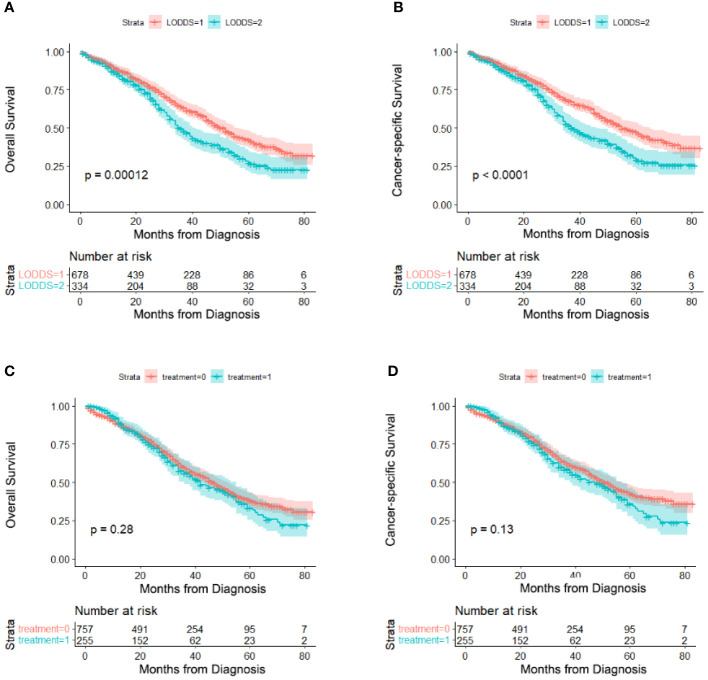
Kaplan–Meier survival curves of LODDS and treatment on OS and CSS. **(A)** LODDS on OS, **(B)** LODDS on CSS (1: LODDS ≤ 0.1, 2: LODDS>0.1), **(C)** treatment on OS, **(D)** treatment on CSS (0: PCS, 1: NACT-ICS).

Kaplan-Meier curves were performed to analyze the effects of LODDS and different treatment (PCS and NACT-ICS) on the OS and CSS of AOC patients with optimal cytoreductive surgery. Significant differences were seen in OS (5-year OS 42.2% vs. 27.2%), and CSS (5-year CSS 47.3% vs. 29.4%) between LODDS ≤0.1 and LODDS >0.1 groups in general (**Figures 2A, B**). However, no significant differences were detected in OS (5-year OS 38.2% vs. 31.7%), and CSS (5-year CSS 42.7% vs. 35.1%) between PCS and NACT-ICS groups (**Figures 2C, D**).

### Predictors for OS and CSS

In the univariable analysis, age, marital status, histology type, tumor size, and LODDS were all associated with OS and CSS. In the predictors of OS, the multivariable Cox regression model showed that patients 60-74 years old (HR:1.378, 95% CI:1.119-1.696) and ≥75 (HR:1.977, 95% CI: 1.480-2.643) had poorer outcomes compared with those 20-59. Additionally, poor outcomes were seen in unmarried patients (HR:1.512, 95%CI: 1.177-1.942), separated, divorced, or widowed (HR:1.318, 95%CI:1.040-1.671), mucinous histology type (HR:2.754, 95% CI: 1.379-5.501), tumor size ≥15 cm (HR:4.179, 95%CI: 3.285-5.315) or LODDS >0.1 (HR: 1.517, 95%CI: 1.238-1.859) (**Table 3**). The multivariable Cox regression model also showed the same results in the predictors of CSS (**Table 3**).

**Table 3:** Multivariable Cox regression of OS and CSS among AOC patients with optimal cytoreductive surgery.

Variable	Multivariable analysis for OS		Multivariable analysis for CSS	
	Hazard ratio (95% CI)	*P*-value*	Hazard ratio (95% CI)	*P*-value*
**Age**				
20-59	1 (Reference)		1 (Reference)	
60-74	1.378 (1.119-1.696)	**0.003**	1.310 (1.050-1.633)	**0.017**
≥75	1.977 (1.480-2.643)	**<0.001**	2.041 (1.507-2.763)	**<0.001**
**Married status**				
Married	1 (Reference)		1 (Reference)	
Unmarried	1.512 (1.177-1.942)	**0.001**	1.447 (1.106-1.893)	**0.007**
Other	1.318 (1.040-1.671)	**0.022**	1.342 (1.046-1.721)	**0.021**
**Histology**				
Serous	1 (Reference)		1 (Reference)	
Endometrioid	1.451 (0.861-2.446)	0.162	1.486 (0.865-2.553)	0.144
Clear cell	1.349 (0.766-2.374)	0.300	1.281 (0.693-2.365)	0.428
Mucinous	2.754 (1.379-5.501)	**0.004**	2.715 (1.303-5.659)	**0.008**
Other	1.309 (0.992-1.728)	0.057	1.108 (0.814-1.509)	0.529
**Tumor size**				
<15	1 (Reference)		1 (Reference)	
≥15	4.179 (3.285-5.315)	**<0.001**	4.396 (3.420-5.686)	**<0.001**
**LODDS**				
≤0.1	1 (Reference)		1 (Reference)	
>0.1	1.517 (1.238-1.859)	**<0.001**	1.553 (1.245-1.911)	**<0.001**

*Bold P-value, P < 0.05.

### Subgroup Analysis of the Effects of LODDS on OS and CSS

**Figure 3:**
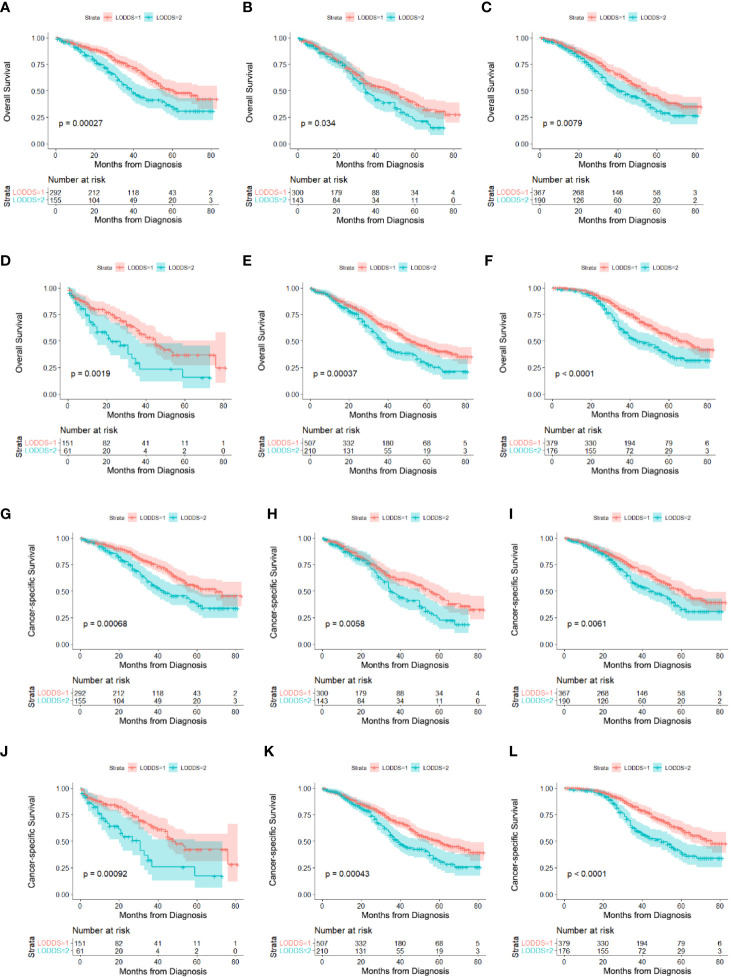
Survival comparison in subgroups of AOC patients with optimal cytoreductive surgery based on LODDS. **(A)** 20-59 years old on OS, **(B)** 60-74 years old on OS, **(C)** married on OS, **(D)** unmarried on OS, **(E)** FIGOIII on OS, **(F)** tumor size<15cm on OS, **(G)** 20-59 years old on CSS, **(H)** 60-74 years old on CSS, **(I)** married on CSS, **(J)** unmarried on CSS, **(K)** FIGOIII on CSS, **(L)** tumor size<15cm on CSS,.

According to the log-rank tests and Cox regression analysis, we studied the effects of LODDS on the prognosis of AOC patients with optimal cytoreductive surgery. As shown in **Table 4**, LODDS was found to be an independent prognostic factor of OS in patients 20-59 years old (HR:1.745, 95% CI:1.289-2.364), 60-74 years old (HR:1.363, 95% CI:1.023-1.815), married (HR:1.426, 95% CI:1.095-1.857), unmarried (HR:1.995, 95% CI:1.281-3.107), FIGO III (HR:1.539, 95% CI: 1.212-1.954) or tumor size <15 cm (HR:1.720, 95% CI: 1.311-2.258). The multivariable Cox regression model also showed the same results in the predictors of CSS (**Figures 3A–L**).

**Table 4:** Multivariable analysis for LODDS on OS and CSS based on age, marital status, FIGO stage, tumor size and NACT among AOC patients with optimal cytoreductive surgery.

		Multivariable analysis for OS			Multivariable analysis for CSS	
Variable	5-year OS (%)	Hazard ratio (95% CI)	*P*-value*	5-year CSS (%)	Hazard ratio (95% CI)	*P*-value*
***Age***						
**20-59**						
**LODDS**						
≤0.1	50.1	1 (Reference)		53.9	1 (Reference)	
>0.1	33.7	1.745 (1.289-2.364)	**<0.001**	37.9	1.730 (1.256-2.383)	**0.001**
**60-74**						
**LODDS**						
≤0.1	38.4	1 (Reference)		45.4	1 (Reference)	
>0.1	21.1	1.363 (1.023-1.815)	**0.034**	22.4	1.529 (1.129-2.070)	**0.006**
**≥75**						
**LODDS**						
≤0.1	23.6	1 (Reference)		22.6	1 (Reference)	
>0.1	13.1	1.401 (0.816-2.407)	0.221	13.6	1.443 (0.827-2.519)	0.197
***Marital status***						
**Married**						
**LODDS**						
≤0.1	44.6	1 (Reference)		50.6	1 (Reference)	
>0.1	30.7	1.426 (1.095-1.857)	**0.008**	32.8	1.473 (1.115-1.945)	**0.006**
**Unmarried**						
**LODDS**						
≤0.1	36.9	1 (Reference)		42.2	1 (Reference)	
>0.1	15.6	1.995 (1.281-3.107)	**0.002**	17.1	2.185 (1.362-3.508)	**0.001**
**Other**						
**LODDS**						
≤0.1	35.3	1 (Reference)		39.6	1 (Reference)	
>0.1	21.8	1.261 (0.847-1.876)	0.253	26.3	1.301 (0.859-1.971)	0.215
***FIGO***						
**III**						
**LODDS**						
≤0.1	44.8	1 (Reference)		50.0	1 (Reference)	
>0.1	27.3	1.539 (1.212-1.954)	**<0.001**	31.2	1.570 (1.219-2.023)	**<0.001**
**IV**						
**LODDS**						
≤0.1	34.5	1 (Reference)		39.5	1 (Reference)	
>0.1	24.9	1.209 (0.870-1.680)	0.259	25.7	1.347 (0.954-1903)	0.091
***Tumor size***						
**<15**						
**LODDS**						
≤0.1	55.7	1 (Reference)		61.6	1 (Reference)	
>0.1	36.9	1.720 (1.311-2.258)	**<0.001**	39.3	1.927 (1.444-2.571)	**<0.001**
**≥15**						
**LODDS**						
≤0.1	23.0	1 (Reference)		27.2	1 (Reference)	
>0.1	0.0	1.236 (0.849-1.799)	0.268	0.0	1.276 (0.860-1.893)	0.226

*Bold P-value, P < 0.05.

## Discussion

Patients with AOC have a poor prognosis and the 5-year survival rate is less than 30% (23). Complete cytoreductive surgery with platinum-based chemotherapy is the standard treatment for patients with AOC (7). The Society of Gynecologic Oncology and American Society of Clinical Oncology issued practice guidelines recommending NACT for patients with high perioperative risk or low likelihood of achieving optimal cytoreductive surgery (5). In our retrospective study, 757 AOC patients with optimal cytoreductive surgery had undergone NACT while 255 did not. Compared with the PCS group, the NACT-ICS group had a higher proportion of older patients, lower PLNs, lower LODDS, multiple sites of metastasis, tumor size ≥15 cm, and FIGO IV. In addition, multivariable logistic regression showed ≥60 years old, married, tumor size ≥15 cm, FIGO IV and LODDS ≤0.1 were associated with higher NACT odds.

Several studies have confirmed that NACT may improve the satisfactory tumor-reducing rate in AOC and suggest that NACT enhances quality of life and lowers the incidence of postoperative adverse events (9, 10). Considering that most patients in the NACT-ICS group were older, later staged, and had poor health, this study believes that NACT is a reasonable treatment option for these AOC patients with difficulty in optimal cytoreductive surgery. However, other studies have found that NACT may increase the risk of platinum resistance and tumor recurrence (24, 25). Our study found NACT was not an independent prognostic factor in AOC patients with optimal cytoreductive surgery and had no significant differences in OS and CSS between PCS and NACT-ICS groups, which agrees with previous studies (10, 12–15).

In general, approximately 60% to 70% of AOC patients with optimal cytoreductive surgery experience peritoneal disease relapse (26). Intraperitoneal chemotherapy when delivered under hyperthermic conditions is termed hyperthermic intraperitoneal chemotherapy (HIPEC), which is a good strategy to address peritoneal disease spread (27). The randomized OVHIPEC study showed that adding HIPEC to ICS improves recurrence-free and OS in patients with stage III OC (28). In addition, the OVHIPEC-2 trial is evaluating HIPEC at PCS and plans to enroll 538 patients with FIGO III epithelial OC (29). Further, HIPEC has been studied in platinum-sensitive recurrent OC patients (30). NCCN guidelines support incorporating this treatment into the counseling of patients considering IDS (1). However, information about whether to use HIPEC was not provided in the SEER database. Combined with the existing data, the research regarding time point, patient selection, drug choice, dose, and duration of HIPEC will be an area of intense research in the future.

The multivariable Cox model showed older (≥60 years old), unmarried, separated, divorced, or widowed, mucinous histology type, tumor size ≥15 cm, or LODDS >0.1 correlated with increased risks of OS and CSS in AOC patients with optimal cytoreductive surgery. Previous studies have shown that marital status is an independent prognostic factor for many cancers (31–33). It is well known that emotional stress down-regulates the cellular immune response and stimulates tumor angiogenesis (34). Separated, divorced, or widowed marital status may limit psychosocial support and personalized care a woman receives. In addition, older patients may have poor health and larger tumors may increase the physical burden, so such patients have lower survival rates. Further, mucinous histology, a rare subtype of epithelial OC, has a poor response to conventional chemotherapy regimens. The early prognosis of this subtype is good, but the prognosis of advanced and recurrent patients is extremely poor (35). Our results showed that LODDS ≤0.1 showed significant survival advantages and higher odds of NACT over patients with LODDS >0.1. We speculate that NACT might be related to a decrease in LODDS. A previous study showed the systematic pelvic and paraaortic lymphadenectomy in AOC patients with normal lymph nodes both before and during surgery was not associated with longer overall or progression-free survival than no lymphadenectomy and was associated with a higher incidence of postoperative complications (36). This suggests that NACT may provide some benefits by reducing the chance of lymphadenectomy. Particularly, stratification analyses were carried out based on several established risk factors for control confounders. LODDS was found to be an independent prognostic factor of OS and CSS in patients <75 years old, married, unmarried, FIGO III and tumor size <15 cm, indicating that LODDS was a meaning prognostic factor. According to these results, clinicians can more reasonably use LODDS grade to predict the prognosis among these patients.

Our research has several limitations. First, information about whether to use HIPEC and regimens and the number of cycles of NACT were insufficient. Second, the SEER database is based on registrations in the United States and whether it applies to other countries or regions is unknown. Despite these inevitable limitations, this study provides an important indication that NACT is not an independent prognostic factor in AOC patients with optimal cytoreductive surgery, but it may contribute to the reduction of LODDS, which is an independent prognostic factor for AOC patients with optimal cytoreductive surgery. And patients with lower LODDS may receive some benefits by reducing the chance of lymphadenectomy.

## Data Availability Statement

All the primary data were acquired from the SEER database (https://seer.cancer.gov/seerstat/).

## Author Contributions

Study concept and design: R-fA. Data acquisition: Y-mH and J-mY. Data analysis and interpretation: Y-mH and YX. Software: YX. Manuscript preparation: Y-mH and FF. Critical revision: R-fA. All authors contributed to the article and approved the submitted version.

## Conflict of Interest

The authors declare that the research was conducted in the absence of any commercial or financial relationships that could be construed as a potential conflict of interest.

## Publisher’s Note

All claims expressed in this article are solely those of the authors and do not necessarily represent those of their affiliated organizations, or those of the publisher, the editors and the reviewers. Any product that may be evaluated in this article, or claim that may be made by its manufacturer, is not guaranteed or endorsed by the publisher.
